# Acquired Penile Epidermoid Cysts in Children

**DOI:** 10.7759/cureus.27462

**Published:** 2022-07-29

**Authors:** Ismail Yagmur, Ali Tekin, Uygar Bağcı, Banu Yaman, Ali Avanoglu, Ibrahim Ulman

**Affiliations:** 1 Department of Pediatric Surgery, Division of Pediatric Urology, Faculty of Medicine, Ege University, Izmir, TUR; 2 Department of Pathology, Faculty of Medicine, Ege University, Izmir, TUR

**Keywords:** penis, epidermoid cyst, hypospadias, circumcision, children

## Abstract

Background

In this study, we aim to present the experience of a tertiary center regarding penile epidermoid cysts over 15 years.

Methodology

Patient files of those who underwent surgical excision for penile epidermoid cysts between 2005 and 2019 were reviewed retrospectively. The demographics, clinical characteristics, etiological factors, cyst features, surgical techniques, complications, and follow-up data were analyzed.

Results

In total, 24 penile epidermoid cysts were excised in 21 boys. The median age at the time of surgery was 52 (15-204) months. The median duration between previous surgery and cyst excision was 40 (1-180) months. In total, 11 cases had a history of circumcision, and 10 had undergone hypospadias surgery. There was no significant difference between these two etiologic groups (p > 0.05). The main symptom was an asymptomatic penile mass. The average cyst size was 9.4 ± 6.7 mm. All cysts were completely excised with incisions made over old scars, except one. No complications were observed during a median follow-up period of 50 (12-120) months, and only one recurrence was noted.

Conclusions

Acquired penile epidermoid cysts may present as an early or late complication after penile surgery. Complete excision with an incision along the existing scars seems to be an effective solution for preventing new scars.

## Introduction

Epidermoid cysts are frequently observed in the face, neck, head, and thoracoabdominal regions of the body. Rarely, they are located in the penis with unknown etiology. They may be congenital or acquired [1]. Congenital epidermoid cysts are believed to occur as a result of abnormal closure of the median raphe during embryogenesis [2,3]. Acquired penile epidermoid cysts (PECs) occur by traumatic implantation of the epidermal components into the dermis, usually as a result of surgical procedures such as circumcision and hypospadias repair [3,4]. Özkan et al. reported the rate of inclusion cyst development after circumcision as 0.015% in a series of 1,900 cases [5].

The literature currently available on PECs consists of case reports. Terms such as epidermoid inclusion cyst, epidermal cyst, epidermal inclusion cyst, infundibular cyst, inclusion cyst, and keratin cyst are used as synonyms of PECs in these case reports [6]. Confusion can arise during literature reviews due to these different terminologies [7].

Prospective or retrospective clinical research articles are lacking when databases were searched for PEC and its synonyms, and data were available as case reports. This suggests either that these cysts are extremely rare or they are underreported. This study aimed to present the demographic characteristics, etiological factors, cyst characteristics, treatment modalities, and follow-up results by retrospectively examining the records of patients who underwent surgical excision due to PECs in our clinic over the last 15 years.

## Materials and methods

Files and computer records of patients who underwent penile cyst/mass excision in the pediatric urology clinic of our hospital were retrospectively reviewed between 2005 and 2019. Etiological factors, age, symptoms, cyst size, location, history of previous penile surgical procedure, date of operation, type of operation, the time between previous penile surgery and cyst excision, postoperative follow-up time, and pathology reports were recorded. The study was approved by the local institutional ethical committee (approval number: 20-1.1T/5).

Surgery was performed for all patients under general anesthesia in the operating room. Except for two cases with multiple cysts, all cysts were solitary and mobile. Except for a case with a cyst at the penopubic junction, all mobile cysts were pushed to the nearest incision scar line, incisions were made over the old scar, and complete excision was performed. The incision was primarily closed. Cyst materials obtained by surgical excision were sampled macroscopically and sections were examined by a pathologist.

A total of 21 boys who underwent surgical excision of penile cysts whose histopathological examination confirmed the presence of epidermoid cyst(s), who were postoperatively followed up for at least one year, and who had a history of penile surgery were included in the study. Cases where histopathological examination did not reveal epidermoid cysts (parameatal cyst, median raphe cyst, granuloma, etc.), those with a follow-up of less than one year, and no history of penile trauma or surgery were excluded from the study.

The patients were divided into two groups according to their history of circumcision and hypospadias surgery. The two groups were compared regarding age, size of the cysts, and time of detecting the cysts. The data obtained were entered into Microsoft Excel 2013 sheet and analyzed using SPSS version 23.0 (IBM Corp., Armonk, NY, USA). Differences were analyzed with independent-sample t-tests. P-values of <0.05 were considered significant.

## Results

Data on the demographic characteristics of 21 boys included in our study and the characteristics of penile cysts are summarized in Table 1.

**Table 1:** Characteristics of patients and penile cysts. P-values of < 0.05 were considered significant.

	Post-circumcision patients	Post-hypospadias repair	P-value
Number of cases	11	10 (six distal, four proximal)	
Number of cysts	11	13	
Average cyst size (mm)	8.2 ± 4.9 (2–20)	10.4 ± 8.0 (2–30)	0.182
Location of penile cysts	Ventral (n = 7), lateral (n = 4)	Ventral (n = 6), lateral (n = 3), dorsal (n = 4)	
Cyst-specific symptoms	Asymptomatic penile mass (n = 7), infected (n = 1), meatal stenosis and mass (n = 1), incidental (n = 2)	Asymptomatic penile mass (n = 5), fistula and mass (n = 3), chordee and mass (n = 1), incidental (n = 1)	
Median age of excision of penile cysts (months)	52 (15–192)	50 (24–204)	0.972
Median age of first surgical intervention (months)	14 (2–84)	18 (9–70)	
Median time between two interventions (months)	40 (1–108)	23 (11–180)	0.307
Median follow-up time (months)	50 (12–120)	54 (12–96)	0.899
Complications	-	-	
Number of recurrences	One	-	
Malignant transformation	-	-	

The median age of our patients was 52 (15-204) months. Among these, 11 patients had a history of circumcision, and 10 had a history of hypospadias surgery. There was no statistically significant difference between the two groups in terms of age (p > 0.05). Proximal hypospadias repair was performed in four patients and distal repair in six. All proximal hypospadias were penoscrotal type.

Complete excision was performed to remove 24 cysts in 21 boys. Single cysts were present in 19 patients, and only two patients with a history of hypospadias surgery had multiple cysts. When cyst localization on the shaft of the penis was evaluated, 13 (54%) were ventral, seven (29%) were lateral, and four (17%) were dorsal. The mean size of the cysts was 9.4 ± 6.7 mm, with a size range of 2-30 mm (median = 8 mm). When the patient groups were compared based on circumcision and hypospadias surgery history, no statistically significant difference was found regarding cyst size (p > 0.05).

We observed that 12 (67%) of 18 patients who presented with swelling in the penis were asymptomatic. In addition to penile swelling, a urethral fistula was present in three patients, meatal stenosis in one patient, and ventral chordee in another patient. Further, one patient with a cyst infection presented with complaints of swelling, inflammation, and pain in the penis. Cysts in three patients were not noticed by their parents; they were detected incidentally by us. All incidentally detected cysts were ≤4 mm in size.

The mean time between the previous surgical procedure and cyst excision was 45.4 ± 42.4 months. This period also includes the time at which the cysts were noticed by the patients or their parents. Whereas the mean time was 48.0 ± 35.0 months in the circumcision group, it was 42.6 ± 51.2 months in the hypospadias group. However, there was no statistically significant difference between the two groups (p > 0.05).

All patients had normal blood and urine test results, except one patient with cyst infection who had mild elevations in white blood cell and C-reactive protein levels.

Pathology results were uniformly reported as epidermoid cysts. All surgeries were performed on an outpatient basis, and no complications were observed in the intraoperative and/or postoperative early period controls. Our median follow-up period was 50 (12-120) months. Recurrence was observed in only one patient during follow-up; however, no malignant transformation was encountered.

## Discussion

Several mechanisms of the formation of epidermoid cysts have been described. Sequestration of epidermal rests during the embryological period, occlusion of the pilosebaceous unit, or the traumatic or surgical implantation of epithelial elements cause the development of these cysts [8]. However, true epidermoid inclusion cysts are formed by the implantation of epithelial elements into the dermis [9]. Acquired PECs can occur on any site of the surgical region. Due to penile skin’s unique properties with less subcutaneous fat and being thinner compared to other locations, we speculate that penile incisions are more likely to develop epidermoid cysts unless appropriate basic skin closure rules are employed. Unlike congenital PECs, acquired PECs do not have to be associated with genitoperineal raphe extending in the midline [10]. In our series, 54% of the penile cysts were ventral, 29% were lateral, and 17% were dorsal. The fact that the cases in our series had a history of penile surgery and that none of the cysts were associated with median raphe support the conclusion that all cases were acquired PECs. Circumcision and hypospadias surgery are the most frequently suspected causes of PECs, and it has been reported that these cysts may occur after surgeries for Peyronie’s disease and penis enlargement [11,12]. Of the 21 cases in our series, 11 had a history of circumcision, and 10 had a history of hypospadias surgery.

It has been reported that PECs may occur in the early or late period after penile surgery [8,13]. While Ofoha et al. presented a case with PEC that occurred a few weeks after circumcision, Ealai et al. reported another case with PEC that presented 10 years after circumcision [8,13]. When the time to notice the cysts in our series was evaluated, the shortest period was one month in a case of circumcision, while the longest period was 180 months (15 years) in a case of hypospadias.

**Figure 1:**
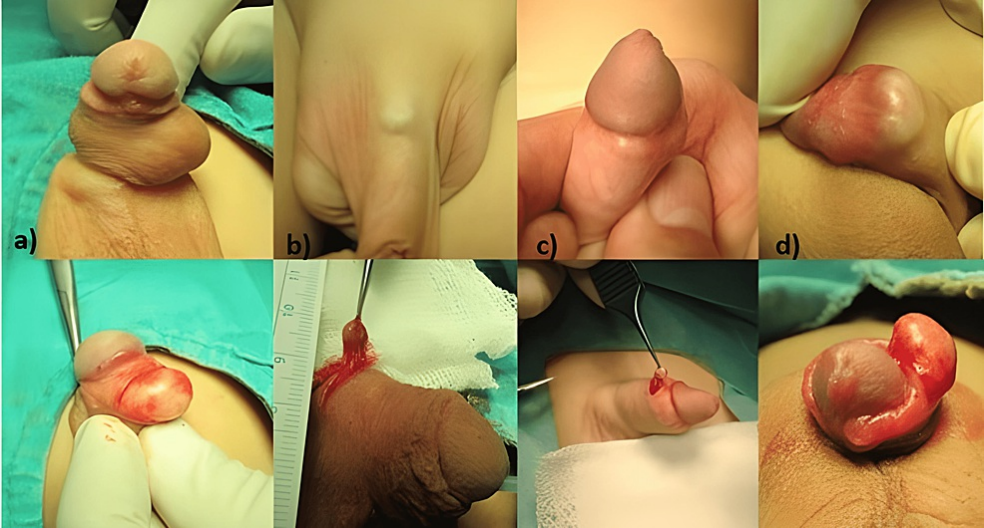
Epidermoid cysts in different locations of the penis. Epidermoid cysts in different locations of the penis and incisions made to remove these cysts. (a) a cyst in the body of the penis, (b) a cyst in the penopubic junction, (c) a cyst in the coronal sulcus, and (d) a cyst at the subcoronal level.

Recent case reports have reported that epidermoid cysts occur even after procedures such as needle biopsies and acupuncture that cause minor trauma [9,14]. It has been suggested that these practices lead to the formation of cysts by causing traumatic implantation of the epidermis and occlusion of eccrine sweat glands and hair follicles [4,7,14]. In our series, in a case with a history of hypospadias surgery, we believe that cyst formation occurred due to penile block injection used for postoperative analgesia because the cyst was in the penile dorsum close to the penopubic junction (Figure 1, Panel b).

PECs are rigid, solitary, well-circumscribed, round, painless, and mobile masses that generally grow slowly [3,4,15,16]. Diagnosis is usually made using clinical findings and confirmed by histopathological examination. Magnetic resonance imaging (MRI) may be useful to show the anatomical borders of the lesion in cysts that are large and extend into the pelvis [15]. However, additional imaging methods are not recommended for every case [16,17]. In our series, we did not use MRI because all of our cases were in the pediatric age group and the cysts were not very large.

Characteristically, PECs tend to grow gradually over time and can reach large sizes [3]. Chen et al. reported an adult case with an 8 × 3 × 3 cm epidermoid cyst located in the penis [16]. Similar to the case report presented by Saini et al., Cankorkmaz et al. also presented a case of a five-year-old boy with a 3 × 2 cm penile epidermoid cyst that developed after circumcision. These cases had larger cysts than other pediatric cases in the literature [9,17]. In our series, the largest cyst was 30 mm in size, and the average cyst size was 9.4 ± 6.7 mm. Incidentally detected cysts were ≤4 mm in size. Therefore, PECs can present with a broad range of size distribution, ranging from large cysts that may cause undesirable cosmetic and functional results to small cysts that are not noticed by the parents but can only be detected incidentally by physicians.

Indications for PEC excision surgery include the development of secondary infection, the tendency of the cyst to grow, obstruction of the urinary tract (especially in large cysts), cosmetic reasons, family decisions, and difficulty during coitus in adult cases [3,4,11,15,16]. The potential for gradual enlargement over time is another indication for removal. Family decisions and cosmetic reasons were the indications for the majority of the interventions in our series. Secondary infection was the indication in one case.

Simple complete excision followed by primary skin closure is generally considered the best treatment modality. After the skin incision is made, a meticulous dissection should be done, and the cyst should be removed completely without opening the capsule [15]. There is a risk of recurrence if aspiration and simple drainage are applied to the cyst [3,4,11,15,18]. Complete excision was performed in all cases in our series. A second excision was required in only one case, five years after excision, due to the development of a new cyst in a different location of the penis.

Acquired epidermoid cysts are mostly mobile masses. This enables the cyst to be easily pushed to the existing scar line, where it is the closest during excision, and thus removed, with the incision made over the old incision scar. In this way, a new scar formation can be prevented. In our series, we preferred this approach to cysts over existing incision scars in almost all cases (Figure 1, Panel a, c, d). We made a new incision only in one case with a cyst at the penopubic junction (Figure 1, Panel b). In the cases presented in this study, the cysts accompanied by urethral fistula, obstruction, penile chordee, and recurrent hypospadias were also excised simultaneously with correction surgeries.

**Figure 2:**
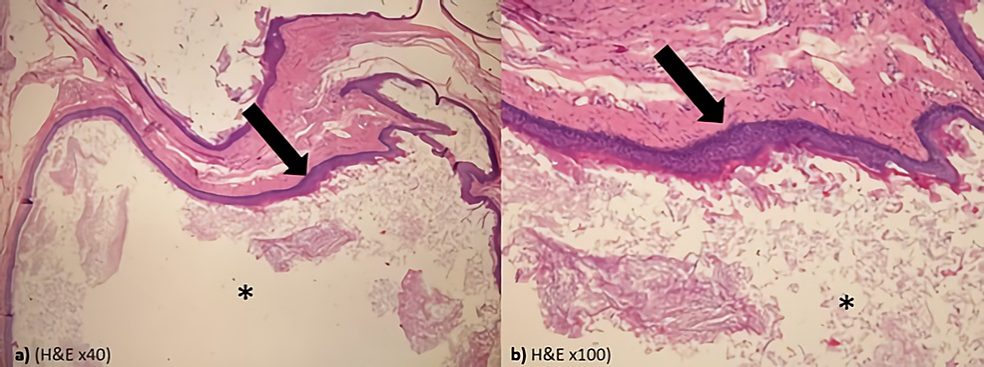
Histopathological imaging of epidermoid cyst. Histologic examination shows a cystic cavity filled with keratin (asterisk) lined by a stratified squamous epithelium that includes a granular layer (arrow). (a) Hematoxylin and eosin ×40; (b) hematoxylin and eosin ×100.

Histopathologically, these cysts consist of a keratin-filled lumen covered by an epithelial cell wall. This epithelium is a stratified squamous epithelium similar to the epidermis and contains a granular layer [19]. Additionally, it should not contain skin appendages and germ cells [20]. Histopathological examination of the cysts excised from the patients in our series also revealed cysts that did not contain germ cells, they were covered with stratified squamous epithelium and filled with keratin (Figure 2).

Although most epidermoid cysts are benign masses that can be treated with simple complete excision, it has been reported that infiltration and recurrence may develop in a minority of cases, and, rarely, even malignant transformation may occur [21-23]. In our series, the follow-up period was 50 (12-120) months, and we did not find any signs of malignant transformation during this period.

The limitations of this study include its retrospective design, absence of a control group, and small sample size.

## Conclusions

PECs may present as an early or late complication after penile surgeries such as circumcision and hypospadias. It should be kept in mind that these benign cysts, which are not symptomatic unless they are infected, can be detected incidentally. Complete excision is a successful treatment modality with a low recurrence rate. Making the incision along the existing incisional scars prevents the formation of new scars and produces more acceptable results. The incidence and etiological factors of PEC can be revealed with future multicenter, prospective studies with larger patient groups.
